# Financial precarity, food insecurity, and psychological distress prospectively linked with use of potentially dangerous dietary supplements during the pandemic in the US

**DOI:** 10.3389/fpubh.2023.1120942

**Published:** 2023-03-02

**Authors:** S. Bryn Austin, Ariel L. Beccia, Amanda Raffoul, Destiny A. Jackson, Vishnudas Sarda, Jaime E. Hart, Jorge E. Chavarro, Janet Rich-Edwards

**Affiliations:** ^1^Division of Adolescent/Young Adult Medicine, Boston Children's Hospital, Boston, MA, United States; ^2^Department of Pediatrics, Harvard Medical School, Boston, MA, United States; ^3^Department of Social and Behavioral Sciences, Harvard T.H. Chan School of Public Health, Boston, MA, United States; ^4^Department of Environmental Health, Harvard T.H. Chan School of Public Health, Boston, MA, United States; ^5^Channing Division of Network Medicine, Department of Medicine, Brigham and Women's Hospital and Harvard Medical School, Boston, MA, United States; ^6^Department of Epidemiology, Harvard T.H. Chan School of Public Health, Boston, MA, United States; ^7^Department of Nutrition, Harvard T.H. Chan School of Public Health, Boston, MA, United States; ^8^Division of Women's Health, Department of Medicine, Brigham and Women's Hospital and Harvard Medical School, Boston, MA, United States

**Keywords:** food insecurity, financial precarity, psychological distress, dietary supplements, COVID-19 pandemic

## Abstract

**Introduction:**

Supplements sold with claims to promote weight loss, cleansing/detoxing, increased energy, or boosted immunity can be dangerous, and consumers experiencing extreme stressors may be especially vulnerable to deceptive claims. The purpose of our study was to investigate associations of financial strain and psychological distress during the COVID-19 pandemic with use of supplements sold for weight loss, cleanse/detox, energy, or immunity.

**Methods:**

We used repeated-measures data gathered over five survey waves from April/May 2020–April 2021 from the COVID-19 Substudy (*N* = 54,951), within three prospective US national cohorts (Nurses' Health Study 2, Nurses' Health Study 3, and Growing Up Today Study), to investigate longitudinal associations between financial strain and psychological distress and risk of use of potentially dangerous types of supplements. Surveys assessed use of supplements prior to and during the first year of the pandemic, as well as financial precarity, food insecurity, depressive and anxiety symptoms, perceived stress, and daily hassles. We fit sociodemographic-adjusted modified Poisson GEE models to estimate risk ratios (RRs) and 95% confidence intervals (CIs) for associations between baseline or lagged time-varying predictors and prevalent or incident (i.e., new-onset) use of each supplement type.

**Results:**

At baseline in April/May 2020, soon after pandemic onset, current use of supplement types was: weight loss 2.7%; cleanse/detox 3.2%; energy 4.4%; immune 22.6%. By the end of the study period, cumulative incidence was: weight loss 3.5%; cleanse/detox 3.7%; energy 4.5%; immune 21.3%. In prevalent-use analyses, financial precarity, food insecurity, and psychological distress were associated with up to 2.4 times the risk of use of these types of supplements across the study period. Similarly, in incident-use analyses, financial precarity and psychological distress were associated with up to 2.1 times the risk of initiating use; whereas, high food insecurity was associated with nearly 1.8 times higher risk of onset of weight-loss supplements use but was not associated with onset of use of other types of supplements.

**Discussion:**

We found consistent evidence that during the first year of the pandemic, participants experiencing elevated financial strain and psychological distress were at heightened risk of initiating use of potentially dangerous types of supplements. Our findings raise concerns about deceptive claims about the safety and product effectiveness by manufacturers of these supplements to profit from vulnerable consumers during the pandemic.

## Introduction

The US market in dietary supplements is massive and fast growing, with over half of adults (1) and nearly a third of children (2) having taken supplements in the past month (3). With the onset of the COVID-19 pandemic, consumer anxiety about infection risk increased appreciably, and the industry saw rising sales for dietary supplements often marketed with deceptive claims of boosting “wellness” (4, 5). These types of potentially dangerous supplements include those sold with claims to lead to weight loss, cleanse/detox, increased energy, or boosted immunity. These types of supplements are not medically recommended, not effective in producing healthful outcomes, and due to toxic ingredients, many have been linked with serious physical harms. Due to widely documented adulteration of supplements on the US market with illicit and toxic ingredients, these products have been linked with numerous serious physical harms, including cardiovascular events, liver injury, and death (6–10). A recent national study found Latin individuals of all genders make up 36%, and women of all race/ethnicity groups make of 74%, of serious liver injury cases, some requiring transplant, caused by green tea extract, a common liver toxin in weight-loss and energy supplements (9). In addition, commonly used dietary supplements can render ineffective prescription medications, such as those for hormonal contraception or to treat blood clots, HIV/AIDS, or organ transplants, with dire effects (6–13). Yet companies selling these types of supplements persist in deceptive promotion of their products (14), misleading consumers about their safety and effectiveness.

Widespread psychological distress during the pandemic has been documented (15), with an estimated threefold increase in depressive symptoms in US adults within the first few months of the pandemic (16). Marketing of dietary supplements often uses positive framing of consumer agency and self-care (17) and, both explicitly and implicitly (18), often includes deceptive claims of health protection (19, 20), such as claims to be a healthful and effective way to reduce risk of illness, weight gain, flagging energy, or the effects of aging. It is plausible that the combination of marketing emphasizing consumer agency and self-care and deceptive claims of safety and effectiveness may lead consumers experiencing elevated psychological distress to use these products during the pandemic, especially if they are seeking to bolster a sense of control and protection in the face of intense and unpredictable pandemic-related stressors. Little is known, however, regarding how psychological distress during the pandemic may be associated with risk of using potentially dangerous types of supplements.

Economic shocks during the pandemic both exacerbated psychological distress and reduced access to food and other basic necessities, with households in communities of color disproportionately impacted by pandemic-related financial precarity and food insecurity (21, 22). By April and May of 2020, food insecurity rate nationwide doubled due to the pandemic (23), peaking at an estimated 23% of households (22), and by 2021 food insecurity was estimated to affect 42 million people in the United States (23). A study of US Census Bureau data collected from April–June 2020 found that household income shocks, such as suddenly becoming unemployed or experiencing reduced work hours, were associated with higher risk of depression and anxiety (24). A recent US nationally representative study found job loss to be associated with substantially elevated psychological distress (25). In research conducted before the pandemic with the National Health and Nutrition Examination Survey, prevalence of past-month use of supplements of any type, which could include vitamins or other types of supplements, was lower in food-insecure (36%) compared to food-secure (55%) households, but prevalence of use still exceeded a third of food-insecure households (26). Potentially dangerous types of supplements are purchased in households at all income levels, but the burden of purchasing proportional to household income is two to four times higher for lower-income than higher-income households, as shown in a national study of weight-loss supplements (27). As a result, while it is plausible that lower-income households may reduce purchasing of these products temporarily during times of heightened financial precarity and food insecurity, prior research suggests that many low-income households continue to purchase potentially dangerous types of supplements, perhaps with the commonly held false belief the products are safe and effective in protecting their own and their family's health (28), thus compounding the financial hardship these households face. Yet no studies we are aware of have assessed how financial precarity and food insecurity during the pandemic may be associated with risk of using potentially dangerous types of supplements.

Revenue during the pandemic has grown precipitously for the US dietary supplements industry. In 2020, US revenue increased over prior years by more than $7 billion, representing 14.5% growth in a single year, and amounting to $56 billion in total revenue by year's end (29). By comparison, in the 5 years preceding the pandemic, the industry's growth in the US averaged $2–$2.5 billion/year (29). Industry analysts estimate that growth in the US supplements market is ~$1.4 billion/year higher than it would have been without the COVID-19 pandemic-related surge in sales, and by 2024 analysts predict US supplements revenue to exceed $66 billion (29).

Prior to the pandemic, widespread dietary supplement fraud, defined as fraud perpetrated for economic gain through selling dietary supplements, was already considered an important public health threat in certain sectors of the supplements market (30, 31). For instance, one content analysis of marketing claims of weight-loss and muscle-building supplements found packages promoted on average 6.5 claims, largely relaying deceptive information claiming to reduce weight, body mass index, or fat; cleanse or detox organs; and boost immunity; furthermore, nearly half of the product packages falsely claimed scientific evidence verified these effects (32).

To address gaps in the literature related to use of potentially dangerous dietary supplements during the pandemic, we gathered longitudinal survey data on financial precarity, food insecurity, psychological indicators of distress, and use of supplements sold for weight loss, cleanse/detox, energy, or immunity in the COVID-19 Substudy, embedded within three existing US national prospective cohorts. The purpose of our study was to investigate associations of financial strain and psychological distress during the COVID-19 pandemic with use of these types of supplements. We hypothesized that financial precarity, food insecurity, and psychological distress would be prospectively, positively associated with both persistent use and new-onset use of potentially dangerous types of supplements.

## Materials and methods

### Study design

The present study uses data provided by participants in the COVID-19 Substudy, which is a closed cohort embedded within three US national, ongoing prospective cohorts Nurses' Health Study 2 (NHS2) (33), Nurses' Health Study 3 (NHS3) (34), and Growing Up Today Study (GUTS) (35) cohorts. NHS2 participants were ages 25–42 years old at enrollment in 1989; NHS3 is an open cohort launched in 2010 where participants were aged 19–49 years at enrollment; GUTS participants were aged 9–16 years at enrollment, which was conducted in two phases with the first in 1996 and the second in 2004. All NHS2 and NHS3 were nursing professionals at the time of enrollment, and all GUTS participants are the children of NHS2 participants (34). Eligible participants were those currently enrolled in NHS2/3 and GUTS with an email on record with cohort administrators and who had returned any pending prior questionnaires (see Supplementary Figure 1 for additional exclusions). By combining the three cohorts, the COVID-19 Substudy included 58,612 participants living in all US states and a very large age range from young to middle adulthood through elder years (GUTS *n* = 6,725; NHS3 *n* = 12,323; NHS2 *n* = 39,566). Participant ages in 2021 were: GUTS 26–40 years; NHS3 30–70 years; NHS2 57–74 years.

For the COVID-19 Substudy, the baseline questionnaire was administered in April-May 2020. During the first 4 months, we conducted monthly follow-ups from baseline among all participants. After the first 4 months, we conducted follow-up questionnaires every 3 months through April 2021 for a total of seven study waves administered to all participants (see Supplementary Figure 2, which also details as to when during the study period our variables of interest were assessed). The overall study was approved by the Brigham and Women's Hospital Institutional Review Board (IRB) and the current analyses by the Boston Children's Hospital IRB.

### Analytic sample

For the current study, we excluded participants who reported living outside the United States at any point during the study period (*n* = 820), because individuals living outside the country may have had different pandemic-related experiences given the substantial heterogeneity in COVID-19 response across countries. We then excluded those who had missing data on key covariates (i.e., age, cohort, race/ethnicity, gender identity, and geographic region of residence; *n* = 1,649) and/or the auxiliary variables used in the construction of inverse-probability-of-censoring weights (IPW) (i.e., COVID-19 infection and related symptomology, occupational status, and mental health status; *n* = 1,208). We also excluded those who never provided information on their use of weight-loss, cleanse/detox, energy, or immune supplements (*n* = 417), resulting in a total of 54,951 eligible participants. For analyses of incident use of these types of supplements, eligibility was further restricted to the 43,469 participants who provided outcome information at baseline and each subsequent study wave and reported no use prior to pandemic onset. List-wise deletion was used to handle missingness on each predictor separately, resulting in final analytic sample size ranges (which varied for each supplement type) of 40,906–54,951 for prevalent-use analyses and 32,814–43,469 for incident-use analyses.

### Measures

#### Predictors

*Financial strain* was assessed in two ways: The first was with an original single-item measure of general financial strain administered at baseline only that asked, “Since the pandemic began, how much of a concern is having enough money for essentials like food and clothing or for paying rent or mortgage?” with response options including “extremely,” “moderately,” “somewhat,” or “not at all concerning.” We used this assessment at baseline as a proxy for experiences with financial precarity across survey waves. The second was a two-item measure of food insecurity derived from the validated Household Food Security Survey Module (HFSSM) (36). The items asked participants to rate the frequency with which they worried about and/or experienced food running out before having money to buy more. Following federal guidelines (37), we categorized those who responded “sometimes” or “often” to both items as having high food insecurity, those who responded “sometimes” or “often” to one item as having moderate food insecurity, and those who responded “never” to both items as having low food insecurity. These items were administered once during the follow-up survey administered in July 2020; given research showing that food insecurity tended to track over time within individuals during the first year of the COVID-19 pandemic (38, 39), we used this single assessment as a proxy for experiences with food insecurity across survey waves.

*Indicators of psychological distress* included depressive symptoms, anxiety symptoms, perceived stress, and daily hassles. Depressive and anxiety symptoms were assessed at each study wave using the Patient Health Questionnaire (PHQ-2) (40) and the Generalized Anxiety Disorder Scale (GAD-2) (41), respectively, both of which were operationalized using the validated binary cut-off score of 3 to represent probable presence of symptoms (40, 41). Perceived stress was assessed at most study waves (baseline, May 2020, June 2020, July 2020, and October 2020) using the abbreviated Perceived Stress Scale (PSS-4) (42) and operationalized using a binary indicator contrasting high vs. low levels of perceived stress, where high perceived stress was defined as having a PSS-4 score in the top tertile of the score distribution and low perceived stress was defined as having a score in the lower two tertiles (42). Last-observation-carried-forward imputation was used to fill in values for the two study waves that did not administer the PSS-4 (i.e., January 2021 and April 2021). Finally, daily hassles, defined as experiences in everyday life that an individual finds salient and harmful or threatening to one's own wellbeing, was assessed at baseline only using the Daily Hassles Scale (DHS) (43) and was operationalized in the same manner as the PSS-4 (i.e., using a binary indicator contrasting high vs. low levels of daily hassles, defined using tertiles) (44). We used this assessment at baseline as a proxy for daily hassles experiences across survey waves.

#### Outcomes

We considered use of any of four potentially dangerous types of supplements as outcomes, which were assessed at five of the seven survey waves. Participants were asked at baseline in April/May 2020, “Are you currently using any of the following types of dietary supplements?” with subsequent items asking specifically about use of weight loss, cleanse/detox, energy, and immune supplements. Response options for each supplement type included “No;” “Yes, started before the outbreak;” “Yes; started after the outbreak began.” On follow-up surveys administered in June 2020, October 2020, January 2021, and April 2021, participants were asked if they were currently using each type of supplement. We considered both prevalent use (i.e., current use at a given study wave, regardless of prior use status) and incident use (i.e., new use at a given study wave among those who reported no pre-pandemic use and no prior use at any waves). All outcomes were operationalized as binary and time-varying (i.e., prevalent/incident use vs. no use at a given study wave).

#### Covariates

We identified a parsimonious set of covariates that represented potential confounders of the relationship between the previously listed predictors and use of potentially dangerous types of supplements, including age in years, cohort (NHS2, NHS3, and GUTS), gender identity (cisgender woman, cisgender man, and transgender/gender diverse), race/ethnicity (Asian, Black, Hispanic/Latin, White, and other/unlisted), US geographic region of residence (Midwest, Northeast, South, and West), and current healthcare worker status (yes and no).

### Data analysis

We fit a series of modified Poisson models to estimate risk ratios (RRs) and 95% confidence intervals (CIs) for associations between each predictor and use of each type of supplement. The first set of models included only baseline data to estimate cross-sectional associations between predictors and prevalent supplement use. The second set of models included repeated-measures data from all relevant study waves to estimate longitudinal associations between predictors and prevalent supplement use across the study period. For predictors that were time-varying, we lagged their values by one study wave to ensure correct temporal ordering (i.e., associations between predictor at wave *w*−1 and outcomes at wave *w*), using their baseline values for the unobserved pre-baseline period (45). Finally, the third set of models estimated longitudinal associations between predictors and incident supplement use across the study period among the subset of participants who reported no use at any wave prior to outcome measurement; here, participants were excluded from the analysis after their first report of the outcome (i.e., use of a type of supplement). To account for intra-cluster correlation due to repeated measures, the second and third set of models were fit using generalized estimating equations with robust standard errors and an exchangeable working correlation matrix. All models adjusted for the aforementioned covariates.

Attrition was substantial across the study period (see Supplementary Figure 2: May 2020 19.3%, June 2020 23.8%, July 2020 25.4%, October 2020 27.8%, January 2021 25.0%, and April 2021 17.2%); therefore, we used IPW to re-weight the data such that they reflected the sample composition at baseline. Following the methods outlined by Fewell et al. (45) and expanded upon by VanderWeele et al. (46) for implementing IPW in the context of repeated-measures data, we built weights that predicted censoring at each study wave (for prevalent-use models) or the cumulative probability of remaining uncensored over time (for incident-use models), conditional on demographic characteristics (age, cohort, gender identity, race/ethnicity, geographic region of residence, and healthcare worker status) and a set of auxiliary variables that were associated with loss-to-follow-up and/or item non-response. The auxiliary variables included COVID-19 infection and related symptomology, unemployment, and mental health status. We then incorporated these weights into the longitudinal models described previously such that the resulting estimates could be interpreted as the associations that would have been observed had there been no attrition (assuming no omitted-variable bias). Analyses were conducted in R version 4.1.0 using the *geepack* package (47).

## Results

**Figure 1:**
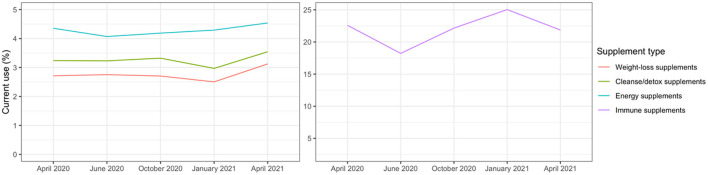
Current use of weight-loss, cleanse/detox, immune, or energy supplements from repeated measures across five waves of the study period (April/May 2020–April 2021) in the COVID-19 pandemic substudy (*N* of participants = 54,277).

Table 1 presents baseline characteristics of the study sample of 54,951 participants living throughout the United States. The sample was largely made up of white cisgender women ranging in age from young adulthood through elder years. At baseline, just under 3% reported experiencing moderate or high food insecurity and slightly more than 6% reported feeling moderately or extremely concerned about having the financial resources to provide household essentials. Depressive (12.9%) and anxiety (21.2%) symptoms were common at baseline, and the prevalence of current use of supplement types was as follows: weight loss 2.7%; cleanse/detox 3.2%; energy 4.4%; and immune 22.6%. Figure 1 depicts the fluctuation in current use of each type of supplement across the five waves, which ranged from lowest to highest as follows: weight-loss 2.7–3.5%; cleanse/detox 3.0–3.5%; energy 4.1–4.5%; immune 18.2–25.0%.

**Table 1:** Baseline sociodemographic characteristics and prevalence of financial stressors and psychological distress by use of weight-loss, cleanse/detox, immune, or energy supplements in the COVID-19 pandemic substudy (*N* = 54,277^a^).

**Characteristics**	**Use of any of four types of supplements^b^ (*n* = 14,427)**	**No use of four types of supplements (*n* = 39,850)**
Age in years, M (SD)	56.2 (13.4)	57.3 (13.6)
Cohort, *n* (%)		
Nurses' Health Study 2	9,523 (66.0)	28,239 (70.9)
Nurses' Health Study 3	3,679 (25.5)	7,696 (19.3)
Growing up today study	1,225 (8.5)	3,915 (9.8)
Gender identity, *n* (%)		
Cisgender women	14,262 (98.9)	39,207 (98.4)
Cisgender men	146 (1.0)	612 (1.5)
Transgender/gender diverse	19 (0.1)	31 (0.1)
Race/ethnicity, *n* (%)		
Asian	187 (1.3)	477 (1.2)
Non-Hispanic Black	243 (1.7)	349 (0.9)
Hispanic/Latin	337 (2.3)	694 (1.7)
Non-Hispanic White	13,280 (92.0)	37,472 (94.0)
Other/unlisted	380 (2.6)	858 (2.2)
Geographic region of residence, *n* (%)		
Midwest	4,158 (28.8)	12,029 (30.2)
Northeast	3,521 (24.4)	11,152 (28.0)
South	3,626 (25.1)	9,168 (23.0)
West	3,122 (21.6)	7,501 (18.8)
Active healthcare worker, *n* (%)	6,597 (45.7)	14,954 (37.5)
Concern over essentials, *n* (%)		
Extremely concerning	331 (2.7)	608 (1.7)
Moderately concerning	610 (5.0)	1,312 (3.7)
Somewhat concerning	2,450 (20.3)	5,767 (16.4)
Not at all concerning	8,700 (72.0)	27,548 (78.2)
Missing (*n*)	2,336	4,615
Food insecurity^c^, *n* (%)		
High	89 (0.9)	157 (0.5)
Moderate	284 (2.8)	545 (1.8)
Low	9,954 (96.4)	29,536 (97.7)
Missing (*n*)	4,100	9,612
Depressive symptoms, *n* (%)	2,101 (14.6)	4,929 (12.4)
Anxiety symptoms, *n* (%)	3,462 (24.0)	8,063 (20.2)
High perceived stress, *n* (%)	705 (4.9)	1,378 (3.5)
Missing (*n*)	2	4
High daily hassles, *n* (%)	887 (7.3)	1,899 (5.4)
Missing (*n*)	2,332	4,602

M, mean; SD, standard deviation. Percentages are column percentages excluding those with missingness (where applicable) and may not sum to 100 due to rounding.

NHS2 participants were born between 1947 and 1964 (ages 25–42 years old at enrollment in 1989); NHS3 is an open cohort launched in 2010; participants enrolled to date were born between 1961 and 1991, making them aged 19–49 years at enrollment; GUTS participants were born between 1981 and 1995 (aged 9–16 years at baseline in 1996 and 2004).

a Excludes those who did not respond to the supplement use items at baseline.

b Weight-loss, cleanse/detox, immune, or energy supplements.

c Assessed during July 2020 follow-up only.

The results of multivariable models estimating cross-sectional associations between financial strain and psychological distress and supplement use at baseline, controlling for confounders, are presented in Table 2. Participants reporting financial strain or psychological distress at baseline were at higher risk of using all four types of supplements at baseline, ranging from 8% to over 180% higher risk.

**Table 2:** Baseline multivariable relative risks (RR) and 95% confidence intervals (CI) for cross-sectional associations between financial stressors, psychological distress, and prevalent use of weight-loss, cleanse/detox, immune, or energy supplements in the COVID-19 pandemic substudy (*N* of participants = 40,906–54,822^a^).

	**Supplement type**			
	**Weight-loss**	**Cleanse/detox**	**Energy**	**Immune**
	**RR (95% CI)**	**RR (95% CI)**	**RR (95% CI)**	**RR (95% CI)**
**Financial stressor**				
**Concern over essentials**				
Extremely concerning	1.88 (1.37, 2.60)	2.00 (1.51, 2.63)	2.18 (1.75, 2.72)	1.41 (1.27, 1.56)
Moderately concerning	1.96 (1.57, 2.46)	1.72 (1.39, 2.13)	2.05 (1.73, 2.43)	1.28 (1.19, 1.39)
Somewhat concerning	1.64 (1.43, 1.87)	1.47 (1.29, 1.66)	1.53 (1.37, 1.71)	1.23 (1.18, 1.28)
Not at all concerning	1.00 (referent)	1.00 (referent)	1.00 (referent)	1.00 (referent)
**Food insecurity**				
High	2.84 (1.79, 4.51)	2.44 (1.59, 3.75)	2.65 (1.88, 3.73)	1.23 (1.01, 1.50)
Moderate	2.32 (1.75, 3.08)	1.68 (1.25, 2.25)	2.21 (1.76, 2.77)	1.20 (1.07, 1.34)
Low	1.00 (referent)	1.00 (referent)	1.00 (referent)	1.00 (referent)
**Psychological distress**				
Depressive symptoms	1.19 (1.04, 1.37)	1.21 (1.06, 1.37)	1.50 (1.36, 1.66)	1.08 (1.04, 1.13)
Anxiety symptoms	1.08 (0.96, 1.22)	1.17 (1.05, 1.31)	1.36 (1.25, 1.49)	1.13 (1.09, 1.17)
High perceived stress	1.39 (1.12, 1.73)	1.27 (1.02, 1.57)	1.50 (1.29, 1.74)	1.17 (1.09, 1.26)
High daily hassles	1.37 (1.12, 1.69)	1.43 (1.19, 1.71)	1.53 (1.31, 1.78)	1.27 (1.19, 1.36)

RR, relative risk; CI, confidence interval. Estimates are derived from modified Poisson models adjusted for age, cohort, gender identity, race/ethnicity, geographic region of residence, and healthcare worker status.

a Analytic N differs by model due to differences in the extent of missingness across predictors; Separate models were examined for each predictor, adjusted for confounders.

Table 3 presents results of multivariable models using repeated measures (*N* of observations = 184,148–222,388, which varied for each supplement type) gathered across the five waves of data collection in the first year of the pandemic, where baseline or lagged indicators of financial strain and psychological distress predicted prevalent use of potentially dangerous types of supplements over the study period and/or at the subsequent wave, respectively. Similar to the cross-sectional models, these longitudinal models indicate that participants reporting financial strain or psychological distress experienced a higher risk of using most types of supplements assessed, ranging from 5% to 140% higher risk. Of note, food insecurity and concern about not having enough money for essentials like food and clothing or for paying rent or mortgage were consistently prospectively associated with elevated risk of using these products.

**Table 3:** Prevalent supplement use: multivariable relative risks (RR) and 95% confidence intervals (CI) for longitudinal associations between financial stressors, psychological distress, and prevalent use of weight-loss, cleanse/detox, immune, or energy supplements from repeated measures across five waves of the study period (April/May 2020–April 2021) in the COVID-19 pandemic substudy (*N* of participants = 41,086–54,951; *N* of observations = 184,148–222,388^a^).

	**Supplement type**			
	**Weight-loss**	**Cleanse/detox**	**Energy**	**Immune**
	**RR (95% CI)**	**RR (95% CI)**	**RR (95% CI)**	**RR (95% CI)**
**Financial stressor**				
**Concern over essentials**				
Extremely concerning	1.84 (1.48, 2.29)	1.60 (1.29, 1.97)	1.86 (1.60, 2.17)	1.39 (1.28, 1.51)
Moderately concerning	1.91 (1.64, 2.23)	1.56 (1.34, 1.82)	1.76 (1.55, 1.99)	1.32 (1.24, 1.40)
Somewhat concerning	1.56 (1.42, 1.72)	1.37 (1.26, 1.50)	1.49 (1.38, 1.61)	1.22 (1.18, 1.26)
Not at all concerning	1.00 (referent)	1.00 (referent)	1.00 (referent)	1.00 (referent)
**Food insecurity**				
High	2.35 (1.64, 3.39)	1.62 (1.09, 2.41)	2.22 (1.70, 2.91)	1.23 (1.03, 1.46)
Moderate	1.70 (1.37, 2.11)	1.33 (1.05, 1.67)	1.63 (1.36, 1.95)	1.25 (1.14, 1.36)
Low	1.00 (referent)	1.00 (referent)	1.00 (referent)	1.00 (referent)
**Psychological distress** ^b^				
Depressive symptoms	1.07 (0.99, 1.16)	1.04 (0.97, 1.12)	1.23 (1.17, 1.30)	1.03 (1.00, 1.05)
Anxiety symptoms	1.02 (0.96, 1.09)	1.04 (0.98, 1.10)	1.12 (1.07, 1.17)	1.05 (1.03, 1.07)
High perceived stress	1.18 (1.03, 1.35)	1.14 (1.00, 1.30)	1.27 (1.15, 1.40)	1.08 (1.03, 1.13)
High daily hassles	1.37 (1.19, 1.58)	1.32 (1.16, 1.52)	1.34 (1.20, 1.49)	1.27 (1.21, 1.34)

RR, relative risk; CI, confidence interval. Estimates are derived from weighted modified Poisson models fitted with generalized estimating equations and adjusted for age, cohort, gender identity, race/ethnicity, geographic region of residence, and healthcare worker status.

a Analytic N differs by model due to differences in the extent of missingness across predictors; Separate models were examined for each predictor, adjusting for confounders; Number of observations describes the number of repeated measures across all participants.

b Depressive symptoms, anxiety symptoms, and perceived stress are time-varying predictors that were analyzed using a one-wave lag with respect to supplement use.

Finally, Table 4 presents results of multivariable models estimating the associations between financial strain and psychological distress with incident use of each type of supplements, restricted to those not previously reporting use of a supplement type. By the end of the study period, cumulative incidence of use post-pandemic onset was: weight loss 3.5%; cleanse/detox 3.7%; energy 4.5%; and immune 21.3%. Participants experiencing financial precarity and psychological distress were at increased risk of beginning to use some types of supplements during the first year of the pandemic. In contrast, high food insecurity was associated with nearly 1.8 times higher risk of onset of weight-loss supplements use but was not associated with onset of use of other types of supplements. The most consistent pattern of risk was observed for participants experiencing concern about not having enough money for essentials, ranging from 21% to 111% elevated risk. Similarly, participants experiencing psychological distress, such as depressive and anxiety symptoms, high perceived stress, and high daily hassles were at increased risk of new use of supplements sold with claims to lead to weight loss, energy, or immune boosting, ranging from 16% to 47% elevated risk.

**Table 4:** New onset supplement use during the pandemic: multivariable relative risks (RR) and 95% confidence intervals (CI) for longitudinal associations between financial stressors, psychological distress, and incident use of weight-loss, cleanse/detox, immune, or energy supplements in the COVID-19 pandemic substudy across five waves of the study period (April/May 2020–April 2021) (*N* of participants = 32,814–43,469, *N* of observations = 118,454–152,499^a^).

	**Supplement type**			
	**Weight-loss**	**Cleanse/detox**	**Energy**	**Immune**
	**RR (95% CI)**	**RR (95% CI)**	**RR (95% CI)**	**RR (95% CI)**
**Cumulative incidence, %** ^**b**^	3.5	3.7	4.5	21.3
**Financial stressor**				
**Concern over essentials**				
Extremely concerning	1.74 (1.26, 2.41)	1.47 (1.04, 2.06)	2.11 (1.63, 2.73)	1.42 (1.22, 1.65)
Moderately concerning	1.49 (1.17, 1.89)	1.54 (1.23, 1.93)	1.46 (1.18, 1.80)	1.25 (1.12, 1.38)
Somewhat concerning	1.35 (1.18, 1.54)	1.25 (1.09, 1.42)	1.36 (1.21, 1.53)	1.21 (1.14, 1.27)
Not at all concerning	1.00 (referent)	1.00 (referent)	1.00 (referent)	1.00 (referent)
**Food insecurity**				
High	1.77 (1.03, 3.06)	0.92 (0.44, 1.96)	1.64 (0.99, 2.70)	0.92 (0.67, 1.28)
Moderate	1.17 (0.80, 1.71)	0.91 (0.60, 1.38)	1.26 (0.91, 1.73)	1.17 (1.00, 1.37)
Low	1.00 (referent)	1.00 (referent)	1.00 (referent)	1.00 (referent)
**Psychological distress** ^c^			
Depressive symptoms	1.31 (1.14, 1.50)	1.11 (0.96, 1.29)	1.46 (1.29, 1.64)	1.17 (1.10, 1.24)
Anxiety symptoms	1.16 (1.03, 1.31)	1.10 (0.98, 1.24)	1.32 (1.19, 1.46)	1.20 (1.15, 1.26)
High perceived stress	1.22 (0.94, 1.58)	0.96 (0.72, 1.28)	1.47 (1.20, 1.80)	1.26 (1.14, 1.41)
High daily hassles	1.39 (1.13, 1.70)	1.15 (0.93, 1.42)	1.47 (1.23, 1.74)	1.34 (1.23, 1.45)

RR, relative risk; CI, confidence interval. Estimates are derived from weighted modified Poisson models fitted with generalized estimating equations and adjusted for age, cohort, gender identity, race/ethnicity, geographic region of residence, and healthcare worker status.

a Analytic N differs by model due to differences in the extent of missingness across predictors; Separate models were examined for each predictor, adjusting for confounders; Number of observations describes the number of repeated measures across all participants.

b Proportion of participants who reported no pre-pandemic supplement use who started using supplements by the end of the study period.

c Depressive symptoms, anxiety symptoms, and perceived stress are time-varying predictors that were analyzed using a one-wave lag with respect to supplement use.

## Discussion

As the COVID-19 pandemic caused widespread psychological distress and financial hardship to millions of Americans (15, 21, 22) the dietary supplements industry enjoyed rapid market growth (29) selling myriad products promising to consumers health protection broadly and specifically claiming to promote weight loss, cleansing/detoxing, energy, and immunity. Given concerns about inadequate consumer protections by government to prevent deceptive claims, we undertook our study to identify predictors of use of potentially dangerous supplement types during the first year of the pandemic. In a large cohort study of nearly 55,000 US adults, we found that participants experiencing heightened financial precarity, food insecurity, and psychological distress were at up to 2.4 times the risk of use of these products throughout the first year of the pandemic. Furthermore, among those who did not previously report use, these same adverse experiences were associated with up to over two times the risk of beginning to use these potentially dangerous products during the first year of the pandemic. To our knowledge, this study is the first to investigate patterns of dietary supplements use associated with financial strain and psychological distress during the pandemic, and our findings raise concerns about industry practices that may exploit vulnerabilities of consumers hardest hit by the pandemic to sell them products that are not only ineffective, but also may put their own and their family's health in greater peril.

Since the passage of the US federal Dietary Supplements Health Education Act (DSHEA) in 1994, the US supplements industry has grown appreciably, earning $56 billion in total revenue from American consumers by the end of 2020 (29). As a direct result of DSHEA, however, the Food and Drug Administration (FDA) is prohibited from requiring rigorous prescreening of supplements for safety or efficacy before they enter the market (48). Growth for the industry has only accelerated since the start of the pandemic (29). Continued industry growth without robust government oversight should raise alarms among medical and public health professionals, given the substantial evidence linking certain types of supplements with serious injury in consumers, including cardiovascular events, liver injury, and death (6–10).

As is now well-documented, the pandemic provoked widespread psychological distress, with a threefold increase in depressive symptoms within just a few months after pandemic onset in the United States (15, 16). Also well-documented were the profound financial shocks that many experienced in the first year of the pandemic, including reduced income and job loss, leading some to extreme financial precarity, food insecurity, and exacerbated psychological distress (24, 25). In fact, within months of the start of the pandemic in the United States, food insecurity had doubled nationally (23), peaking at 23% of households by April/May 2020 (22), and by 2021 an estimated 42 million people across the country were struggling with food insecurity (23). However, no prior studies we are aware of have investigated how these types of widespread adverse experiences during the pandemic may have increased the risk of use of potentially dangerous types of dietary supplements commonly marketed with positive framing of consumer agency and self-care (17, 18) and with deceptive claims to support consumer health (19, 20). Our study findings support our hypotheses that during the first year of the pandemic, consumers experiencing financial precarity, food insecurity, and psychological distress would be at elevated risk of both prevalent use and new onset of use of potentially dangerous dietary supplements sold for weight loss, cleanse/detox, energy, and immunity. It is plausible that for these types of supplements, consumers facing both financial and psychological adversity may have been particularly vulnerable to product marketing that emphasized consumer agency and self-care along with deceptive claims of safety and effectiveness. Perhaps vulnerable consumers sought to bolster a sense of control and protection as a way to cope with intense pandemic-related stressors. Future research will be needed to explore how deceptive marketing may influence consumer motivations to use potentially dangerous types of dietary supplements without evidence of safety or effectiveness, particularly in times of financial precarity and mental distress. In addition, as prior research has documented disproportionate financial burden on lower-income households attributable to purchases of deceptive weight-loss supplements (27), new research is needed to estimate the added financial burden attributable to supplements on households experiencing financial precarity and other pandemic-related adversities.

Our study has several limitations. Our study cohorts are not representative of the US population, as all participants in NHS2 and NHS3 were professional nurses or nursing trainees at the time of enrollment, and GUTS participants are all children of NHS2 participants. As a result, communities of color, men, and low-income communities are underrepresented in the cohorts. In addition, while both high or moderate food insecurity were positively associated with prevalent use of potentially dangerous types of supplements in the first year of the pandemic, in incident-use analyses, only high food insecurity was associated with new-onset use and only for weight-loss supplements. As only 1,075 participants reported food insecurity at baseline, it is possible that food insecurity is not associated with new-onset use of other types of supplements or that our analyses were not sufficiently powered to detect an association. Our assessment of use of potentially dangerous types of dietary supplements is self-reported, and we do not have information on dose or frequency of use or brand of supplement used, reducing precision in our outcome measurement. Unlike other primary predictors, general financial strain, food insecurity, and daily hassles were assessed at only one time point (so had to be treated as time invariant) and with brief measures that cannot fully capture the complexity of each construct, increasing the likelihood of misclassification. Sample attrition after April/May 2020 was substantial, and it is plausible that bias was introduced if participants most severely affected by the pandemic stopped completing surveys at a higher rate than other participants. In response, we implemented IPW, which is considered the most rigorous analytic approach to mitigate the effects of attrition bias on study findings.

The US dietary supplements industry achieved an unprecedented 14.5% revenue growth during the first year of the pandemic, reaching $56 billion in total revenue by the end of 2020 (29). Our finding that people experiencing heightened financial precarity, food insecurity, and psychological distress during the first year of the pandemic were at greatest risk of using potentially dangerous supplements provides disturbing evidence that companies selling these products profited directly from vulnerable members of our communities. Our findings also amplify ongoing concerns about consumer safety, given the persistent problems of deceptive advertising (4, 5, 32) and widespread consumer misconception that these products are safe and effective (28).

Our findings have important implications for clinicians, public health nutrition professionals, and government to protect consumers from the growing problem of predatory and potentially dangerous supplements. Clinicians should routinely query patients about their use of these types of supplements and counsel them as to the risks. Public health nutrition professionals working with individuals and households experiencing food insecurity and financial precarity should be aware that, despite the expense, these clients may use these products, perhaps with the mistaken belief promoted by industry that the products will protect themselves or their families from illness. Large-scale public health surveillance surveys designed to assess nutrition or risk behaviors should also assess dietary supplement use, particularly the types of potentially dangerous supplements as addressed in our study. Furthermore, descriptive and analytic epidemiologic studies are needed to build on our findings to help illuminate other determinants of use of potentially dangerous supplements and possible leverage points for preventive interventions. Finally, the FDA and Federal Trade Commission have a clear responsibility to take aggressive action against companies employing deceptive advertising and tainting their products with dangerous and illegal ingredients.

Given the myriad serious health risks (6–10) presented by supplements sold with claims to promote weight loss, cleanse/detox, energy, and immunity, our study underscores the compelling need for effective intervention. As the industry continues to capitalize on the COVID-19 pandemic to accelerate the pace and scale of its dangerous products, the need is urgent for a robust and evidence-based public health response to mitigate consumer harm linked with these deceptive products.

## Data availability statement

The raw data supporting the conclusions of this article will be made available by the authors, without undue reservation.

## Ethics statement

The studies involving human participants were reviewed and approved by Brigham and Women's Hospital. The patients/participants provided their written informed consent to participate in this study.

## Author contributions

SA, AB, and AR conceived of the study. AB and VS carried out data analyses. SA and AB produced the initial draft of the manuscript. AR, DJ, VS, JR-E, JC, and JH provided feedback on data analyses and interpretation and critical revisions to the manuscript. All authors contributed to the article and approved the submitted version.
