# Supplementary material for: Financial precarity, food insecurity, and psychological distress prospectively linked with use of potentially dangerous dietary supplements during the pandemic in the US
Source: Front Public Health. 2023 Mar 2;11:1120942. doi: 10.3389/fpubh.2023.1120942 (PMC10018192; doi:10.3389/fpubh.2023.1120942)

**Supplemental Figure 1**. Study flow diagram for recruitment into the COVID-19 Substudy from the Nurses’ Health Study 2 (NHS2), Nurses’ Health Study 3 (NHS3), and Growing up Today Study (GUTS) cohorts.

**19,094 Invited to COVID-19 Substudy**

(11,466 female; 7628 male)

**NHS2:**

116,429

**NHS3:**

35,852

**GUTS:**

27,793

**59,925 Invited to COVID-19 Substudy**

(all female)

**39,564 Responded**

(all female)

**6,725 Responded**

(4,836 female; 1,889 male)

**5,844** died

**4,868** no longer in cohort

**26,610** no email on record

**585** paper questionnaires only

**21,076** main cohort questionnaire pending

**369** opted out all sub-studies

**1,104** currently in another sub-study

**48** self-reported dementia

**40** no longer in cohort

**2,135** no email on record

**3,034** with <2 main questionnaires

**146** died

**1,633** no longer in cohort

**6,920** no email on record

**12,323 Responded**

(12,114 female; 209 male)

**30,643 Invited to COVID-19 Substudy**

(30,123 female; 420 male)

**Supplemental Figure 2**. Study flow diagram for retention within the COVID-19 Substudy and timing of variable assessment.


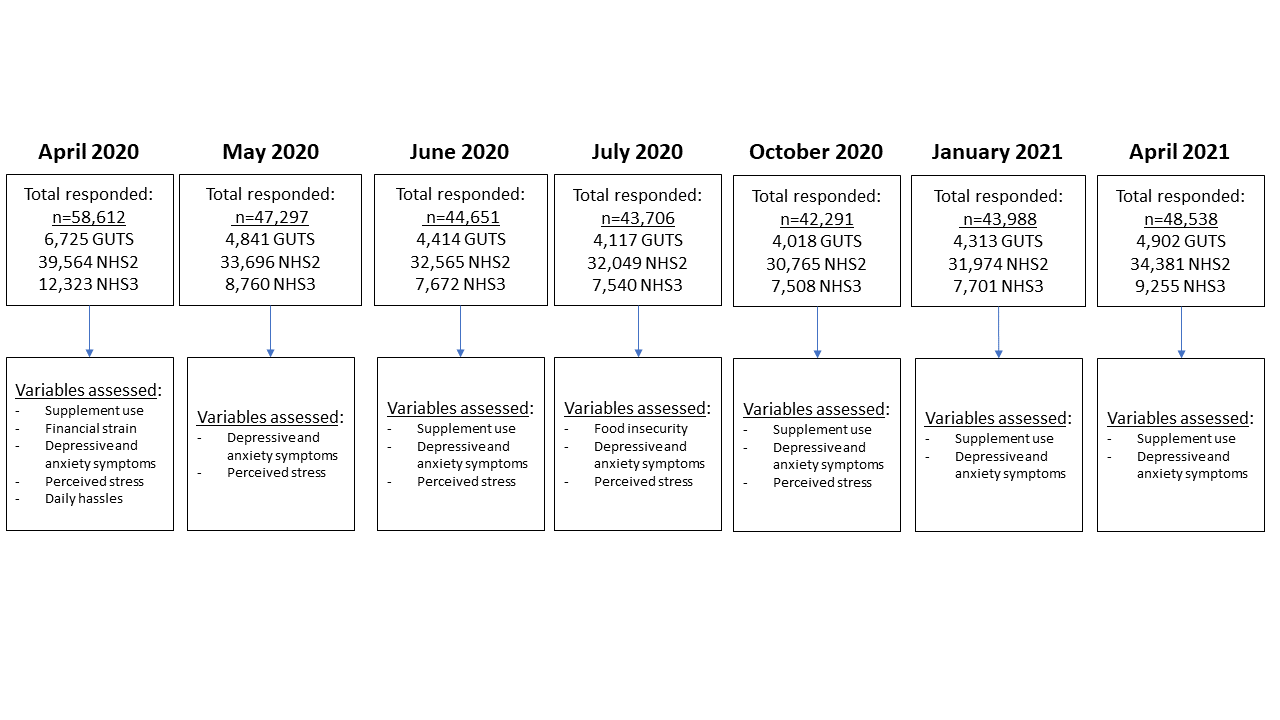

Supplement: Supplementary file 1 [file Data_Sheet_1.docx]
